# Depressive Symptoms and Anxiety During the COVID-19 Pandemic: Large, Longitudinal, Cross-sectional Survey

**DOI:** 10.2196/33585

**Published:** 2022-02-10

**Authors:** James J MacDonald, Ryan Baxter-King, Lynn Vavreck, Arash Naeim, Neil Wenger, Karen Sepucha, Annette L Stanton

**Affiliations:** 1 Department of Psychology University of California, Los Angeles Los Angeles, CA United States; 2 Department of Political Science College of Letters and Sciences University of California, Los Angeles Los Angeles, CA United States; 3 Department of Communication College of Letters and Sciences University of California, Los Angeles Los Angeles, CA United States; 4 Center for SMART Health Clinical and Translational Science Institute University of California, Los Angeles Los Angeles, CA United States; 5 Division of General Internal Medicine and Health Sciences Research David Geffen School of Medicine University of California, Los Angeles Los Angeles, CA United States; 6 Health Decision Sciences Center Massachusetts General Hospital Harvard Medical School Boston, MA United States; 7 Department of Psychiatry and Biobehavioral Sciences University of California, Los Angeles Los Angeles, CA United States

**Keywords:** COVID-19, depression, anxiety, pandemic, mental health, public health, psychological variables, younger adults, symptom monitoring, health intervention

## Abstract

**Background:**

The COVID-19 pandemic has influenced the mental health of millions across the globe. Understanding factors associated with depressive symptoms and anxiety across 12 months of the pandemic can help identify groups at higher risk and psychological processes that can be targeted to mitigate the long-term mental health impact of the pandemic.

**Objective:**

This study aims to determine sociodemographic features, COVID-19-specific factors, and general psychological variables associated with depressive symptoms and anxiety over 12 months of the pandemic.

**Methods:**

Nationwide, cross-sectional electronic surveys were implemented in May (n=14,636), July (n=14,936), October (n=14,946), and December (n=15,265) 2020 and March/April 2021 (n=14,557) in the United States. Survey results were weighted to be representative of the US population. The samples were drawn from a market research platform, with a 69% cooperation rate. Surveys assessed depressive symptoms in the past 2 weeks and anxiety in the past week, as well as sociodemographic features; COVID-19 restriction stress, worry, perceived risk, coping strategies, and exposure; intolerance of uncertainty; and loneliness.

**Results:**

Across 12 months, an average of 24% of respondents reported moderate-to-severe depressive symptoms and 32% reported moderate-to-severe anxiety. Of the sociodemographic variables, age was most consistently associated with depressive symptoms and anxiety, with younger adults more likely to report higher levels of those outcomes. Intolerance of uncertainty and loneliness were consistently and strongly associated with the outcomes. Of the COVID-19-specific variables, stress from COVID-19 restrictions, worry about COVID-19, coping behaviors, and having COVID-19 were associated with a higher likelihood of depressive symptoms and anxiety.

**Conclusions:**

Depressive symptoms and anxiety were high in younger adults, adults who reported restriction stress or worry about COVID-19 or who had had COVID-19, and those with intolerance of uncertainty and loneliness. Symptom monitoring as well as early and accessible intervention are recommended.

## Introduction

As a prolonged, multidimensional stressor, COVID-19 has affected global mental health [1,2]. Sociodemographic and psychological correlates of elevated anxiety and depressive symptoms in early 2020 after pandemic onset are well documented [3]; a younger age, female gender, lower income/unemployment, uncertainty intolerance, and loneliness are associated with worse mental health during the pandemic. These findings primarily are from cross-sectional or short-term longitudinal studies (eg, 4-8 weeks) early in the pandemic. Less is known about contributors to mental health across the pandemic and as it wanes in the United States. Accordingly, this study was designed to examine hypothesized contributors to depressive symptoms and anxiety from 5 waves of data collected over 12 months.

The nature of the expected associations of sociodemographic, psychological, and COVID-19-specific variables with mental health outcomes were hypothesized to change from earlier to later phases of the pandemic. We focused on findings that are robust and consistent and are most pertinent to how the population will emerge from the pandemic.

## Methods

### Data Collection

Data were obtained from 5 national online surveys from May 2020 to April 2021 involving a total of 74,340 adults in the University of California, Los Angeles (UCLA) COVID Health and Politics Project, after institutional review board approval (IRB #20-000786). The samples were provided by Lucid, a market research platform. Prior to survey completion, respondents were informed of the following: the name and contact information of the principal investigator, that completion of the survey was voluntary, that the survey would take approximately 15 minutes, that no personally identifiable information would be asked within the survey, that any identifying information in connection with the study would remain confidential, and that the study was being performed to understand the impact of the COVID-19 pandemic on daily life. Project staff set quotas for sample acquisition and generated weights to produce representative samples of the adult US population. The response rate was approximately 69% on average across waves. Additional details regarding sampling and survey methods are available. [4].

### Outcome Variables

Depressive symptoms were assessed using the Patient Health Questionnaire-8 (PHQ-8) [5], which contains 8 of the 9 *Diagnostic and Statistical Manual of Mental Disorders, Fifth Edition* (DSM-5), major depressive disorder (MDD) symptom criteria. Scores ranged from 0 to 24. Based on recommended cut-offs [5], severity categories were no significant symptoms (0-4), mild symptoms (5-9), and moderate-to-severe symptoms (10).

The 4-item Patient-Reported Outcome Measurement Information System (PROMIS) short form [6] assessed anxiety. Total scores ranged from 4 to 20. Following PROMIS scoring guidelines and established severity cut-offs, raw scores were converted to T scores, and established cut-off points yielded 3 categories: normal, mild, and moderate-to-severe anxiety.

### Independent Variables

Table 1 displays categorical sociodemographic, COVID-19-related, and psychological variables. All independent variables were coded as categorical variables for inclusion in logistic regressions. Respondents were asked to indicate their age, gender (male or female), race/ethnicity, education level, household income, living status, presence of children in the home, employment status in the past 2 months prior to assessment, political identification, and health status (eg, presence or absence of a “significant medical problem or ailment,” including heart disease, cancer, or diabetes). Respondents’ geographical region and urban/rural living status were determined using the respondents’ zip code. Levels of categorical sociodemographic variables and referent categories are displayed in Table 1.

**Table 1:** Characteristics of survey respondents.

Variable level		Weighted percentage, %					
		Wave 1 (N=14,636)	Wave 2 (N=14,936)	Wave 3 (N=14,946)	Wave 4 (N=15,265)	Wave 5 (N=14,557)	Overall (N=74,340)
**Age (years), n (%)**							
	18-29	2975 (20.3)	3036 (20.3)	3046 (20.4)	3100 (20.3)	2958 (20.3)	15,115 (20.3)
	30-44	3703 (25.3)	3798 (25.4)	3911 (26.2)	3866 (25.3)	3922 (26.9)	19,119 (25.8)
	45-64	4953 (33.8)	5106 (34.2)	5054 (33.8)	5114 (33.5)	4925 (33.8)	25,151 (33.8)
	65+^a^	3006 (20.5)	2996 (20.1)	2936 (19.6)	3185 (20.9)	2752 (18.9)	14,875 (20.0)
**Gender, n (%)**							
	Male	7067 (48.3)	7211 (48.3)	7225 (48.3)	7367 (48.3)	7028 (48.3)	35,898 (48.3)
	Female^a^	7569 (51.7)	7725 (51.7)	7721 (51.7)	7898 (51.7)	7529 (51.7)	38,443 (51.7)
**Race/ethnicity, n (%)**							
	White^a^	9266 (63.3)	9456 (63.3)	9512 (63.6)	9666 (63.3)	9222 (63.3)	47,122 (63.4)
	Black	1641 (11.2)	1670 (11.2)	1638 (11.0)	1736 (11.4)	1633 (11.2)	8318 (11.2)
	Asian	8318 (6.9)	1029 (6.9)	1032 (6.9)	1051 (6.9)	1003 (6.9)	5123 (6.9)
	Hispanic	2264 (15.5)	2325 (15.6)	2292 (15.3)	2338 (15.3)	2256 (15.5)	11,475 (15.4)
	Other	457 (3.1)	456 (3.1)	472 (3.2)	475 (3.1)	444 (3.0)	2304 (3.1)
**Education, n (%)**							
	High school or less^a^	4771 (32.6)	4861 (32.5)	4610 (30.8)	5011 (32.8)	4825 (33.1)	24,086 (32.4)
	Some college	5350 (36.6)	5467 (36.6)	5711 (38.2)	5541 (36.3)	5239 (36.0)	27,283 (36.7)
	College and above	4515 (30.8)	4608 (30.8)	4626 (30.9)	4713 (30.9)	4493 (30.9)	22,971 (30.9)
**Heath status, n (%)**							
	Generally healthy^a^	8260 (56.4)	8347 (55.9)	8078 (54.0)	8196 (53.7)	7446 (51.1)	40,326 (54.2)
	Significant diagnosis	6376 (43.6)	6589 (44.1)	6868 (46.0)	7070 (46.3)	7111 (48.9)	34,014 (45.8)
**Household income^b^, n (%)**							
	34,999 or less	2968 (20.3)	3029 (20.3)	3018 (20.2)	3095 (20.3)	2950 (20.3)	15,060 (20.3)
	35,000-79,999	5228 (35.7)	5336 (35.7)	5339 (35.7)	5462 (35.7)	5200 (35.7)	26,565 (35.7)
	80,000 or more	6440 (44.0)	6571 (44.0)	6589 (44.1)	6708 (44.0)	6407 (44.0)	32,715 (44.0)
**Lives alone, n (%)**							
	Yes	2254 (15.4)	2305 (15.4)	2305 (15.4)	2440 (16.0)	2544 (17.5)	11,850 (15.9)
	No^a^	12,347 (84.4)	12,596 (84.3)	12,597 (84.3)	12,720 (83.3)	11,955 (82.1)	62,215 (83.7)
	Missing^c^	34 (0.2)	35 (0.2)	44 (0.3)	105 (0.7)	58 (0.4)	276 (0.4)
**Children living at home, n (%)**							
	Yes	5552 (37.9)	5787 (38.7)	5863 (39.2)	5948 (39.0)	5297 (36.4)	28,447 (38.3)
	No^a^	9013 (61.6)	9088 (60.8)	9012 (60.3)	9187 (60.2)	9189 (63.1)	45,488 (61.2)
	Missing	71 (0.5)	61 (0.4)	72 (0.5)	130 (0.9)	72 (0.5)	376 (0.5)
**Employment in the past 2 months^d^, n (%)**							
	Working in person	3175 (21.7)	4012 (26.9)	4414 (29.5)	N/A^e^	N/A	N/A
	Working remotely	3239 (22.1)	2937 (19.7)	2699 (18.1)	N/A	N/A	N/A
	Not working due to COVID	1974 (13.5)	1555 (10.4)	1364 (9.1)	N/A	N/A	N/A
	Not working for other reason	244 (1.7)	464 (3.1)	489 (3.3)	N/A	N/A	N/A
	Not working prior to COVID^a^	5982 (40.9)	5949 (39.8)	5941 (39.8)	N/A	N/A	N/A
	Missing	21 (0.1)	18 (0.1)	39 (0.3)	N/A	N/A	N/A
**Political identification, n (%)**							
	Democrat	6575 (44.9)	6685 (44.8)	6548 (43.8)	7199 (47.2)	6218 (42.7)	33,225 (44.7)
	Republican^a^	5374 (36.7)	5416 (36.3)	5785 (38.7)	5375 (35.2)	5515 (37.9)	27,465 (36.9)
	Independent	2676 (18.3)	2818 (18.9)	2596 (17.4)	2655 (17.4)	2795 (19.2)	13,540 (18.2)
	Missing	12 (0.1)	17 (0.1)	17 (0.1)	37 (0.2)	29 (0.2)	111 (0.1)
**Region, n (%)**							
	Northeast	2653 (18.1)	2605 (17.4)	2607 (17.4)	2663 (17.4)	2539 (17.4)	13,067 (17.6)
	Midwest	3075 (21.0)	3107 (20.8)	3109 (20.8)	3179 (20.8)	3028 (20.8)	15,499 (20.8)
	South	5695 (38.9)	5664 (37.9)	5668 (37.9)	5786 (37.9)	5521 (37.9)	28,334 (38.1)
	West^a^	3213 (22.0)	3560 (23.8)	3561 (23.8)	3637 (23.8)	3469 (23.8)	17,441 (23.5)
**Urban-rural, n (%)**							
	Rural	3530 (24.1)	3602 (24.1)	3614 (24.2)	3677 (24.1)	3512 (24.1)	17,936 (24.1)
	Suburban	3554 (24.3)	3627 (24.3)	3710 (24.3)	3709 (24.3)	3535 (24.3)	18,054 (24.3)
	Urban-suburban	6183 (42.2)	6309 (42.2)	6311 (42.2)	6450 (42.2)	6149 (42.2)	31,401 (42.2)
	Urban^a^	1369 (9.4)	1397 (9.4)	1393 (9.3)	1428 (9.4)	1361 (9.3)	6949 (9.4)
**COVID-19 infection in the past 2 months, n (%)**							
	Believes no exposure^a^	13,348 (91.2)	13,403 (89.7)	13,409 (89.7)	12,989 (85.1)	12,039 (82.7)	65,188 (87.7)
	Tested positive for COVID-19	209 (1.4)	447 (3.0)	553 (3.7)	794 (5.2)	1211 (8.3)	3213 (4.3)
	Believes had COVID-19	802 (5.5)	730 (4.9)	687 (4.6)	960 (6.3)	651 (4.5)	3831 (5.2)
	Believes household had COVID-19 (but not self)	250 (1.7)	323 (2.2)	271 (1.8)	462 (3.0)	620 (4.3)	1926 (2.6)
	Missing	27 (0.2)	32 (0.2)	27 (0.2)	60 (0.4)	36 (0.2)	182 (0.2)
**COVID-19 restriction stress in the past 2 weeks, n (%)**							
	Not at all^a^	4145 (28.3)	4666 (31.2)	5402 (36.1)	4904 (32.1)	6231 (42.8)	25,348 (34.1)
	Slightly	5389 (36.8)	5386 (36.1)	5085 (34.0)	5126 (33.6)	4227 (29.0)	25,213 (33.9)
	Moderately	2992 (20.4)	2890 (19.3)	2724 (18.2)	3022 (19.8)	2414 (16.6)	14,041 (18.9)
	Very	1354 (9.3)	1227 (8.2)	988 (6.6)	1319 (8.6)	909 (6.2)	5797 (7.8)
	Extremely	718 (4.9)	735 (4.9)	734 (4.9)	843 (5.5)	712 (4.9)	3742 (5.0)
	Missing	38 (0.3)	33 (0.2)	13 (0.1)	50 (0.3)	65 (0.4)	199 (0.3)
**COVID-19 worry in the past month, n (%)**							
	Not worried^a^	3982 (27.2)	3856 (25.8)	4234 (28.3)	3965 (26.0)	5586 (38.4)	21,623 (29.1)
	Mild	5912 (40.4)	6057 (40.6)	6118 (40.9)	6188 (40.5)	5036 (34.6)	29,311 (39.4)
	Moderate-severe	3843 (26.3)	4098 (27.4)	3708 (24.8)	4220 (27.6)	3204 (22.0)	19,073 (25.7)
	Missing	899 (6.1)	925 (6.2)	887 (5.9)	892 (5.8)	731 (5.0)	4333 (5.8)
**COVID-19 risk in the next 30 days, n (%)**							
	Very low^a^	4736 (32.4)	4680 (31.3)	4915 (32.9)	5496 (36.0)	6352 (43.6)	26,180 (35.2)
	Moderately low	4114 (28.1)	4193 (28.1)	4078 (27.3)	3804 (24.9)	3595 (24.7)	19,785 (26.6)
	Neither high nor low	4081 (27.9)	4241 (28.4)	4178 (28.0)	4150 (27.2)	3212 (22.1)	19,862 (26.7)
	Moderately or very high	1686 (11.5)	1797 (12.0)	1761 (11.8)	1796 (11.8)	1375 (9.4)	8414 (11.3)
	Missing	19 (0.1)	25 (0.2)	14 (0.1)	19 (0.1)	22 (0.2)	100 (0.1)
**COVID-19 deaths per 1000 in the past 14 days (terciles)^f^, n (%)**							
	Low density of deaths	4862 (33.2)	4942 (33.1)	4959 (33.2)	5014 (32.8)	4851 (33.3)	24,628 (33.1)
	Medium density of deaths	4827 (33.0)	5011 (33.5)	4990 (33.4)	5176 (33.9)	4860 (33.4)	24,864 (33.5)
	High density of deaths	4947 (33.8)	4983 (33.4)	4997 (33.4)	5075 (33.2)	4846 (33.3)	24,848 (33.4)
**Known COVID-19 deaths^g^, n (%)**							
	0^a^	N/A	N/A	N/A	N/A	8046 (55.3)	N/A
	1	N/A	N/A	N/A	N/A	2277 (15.6)	N/A
	≥2	N/A	N/A	N/A	N/A	4141 (28.4)	N/A
	Missing	N/A	N/A	N/A	N/A	93 (0.6)	N/A
**Vaccination status^g^, n (%)**							
	Fully vaccinated	N/A	N/A	N/A	N/A	4745 (32.6)	N/A
	Partially vaccinated	N/A	N/A	N/A	N/A	2355 (16.2)	N/A
	Not vaccinated^a^	N/A	N/A	N/A	N/A	7457 (51.2)	N/A
**Loneliness, n (%)**							
	No loneliness^a^	9211 (62.9)	9204 (61.6)	9162 (61.3)	9045 (59.2)	8909 (61.2)	45,531 (61.3)
	Any loneliness	5271 (36.0)	5577 (37.3)	5611 (37.5)	6024 (39.5)	5281 (36.3)	27,764 (37.3)
	Missing	153 (1.0)	155 (1.0)	173 (1.2)	197 (1.3)	367 (2.5)	1045 (1.4)
**Uncertainty tolerance, n (%)**							
	High tolerance^a^	5957 (40.7)	6089 (40.8)	6064 (40.6)	6042 (39.6)	6024 (41.4)	30,175 (40.6)
	Medium tolerance	5230 (35.7)	5226 (35.0)	5262 (35.2)	5532 (36.2)	4987 (34.3)	26,238 (35.3)
	Low tolerance	3112 (21.3)	3351 (22.4)	3281 (21.9)	3451 (22.6)	3308 (22.7)	16,503 (22.2)
	Missing	337 (2.3)	270 (1.8)	339 (2.3)	241 (1.6)	238 (1.6)	1424 (1.9)
**Avoidance coping (past 2 weeks), n (%)**							
	No avoidance^a^	7917 (54.1)	8475 (56.7)	8795 (58.8)	8663 (56.8)	9103 (62.5)	42,954 (57.8)
	Any avoidance	6719 (45.9)	6461 (43.3)	6151 (41.2)	6602 (43.2)	5454 (37.5)	31,387 (42.2)
**Approach coping (past 2 weeks), n (%)**							
	Low approach	5177 (35.4)	5476 (36.7)	6134 (41.0)	6022 (39.4)	7205 (49.5)	30,015 (40.4)
	Moderate approach	4116 (28.1)	4222 (28.3)	4175 (27.9)	4464 (29.2)	3804 (26.1)	20,781 (28.0)
	High approach^a^	5343 (36.5)	5238 (35.1)	4637 (31.0)	5780 (31.3)	3547 (24.4)	23,545 (31.7)
**Anxiety (past 7 days), n (%)**							
	No anxiety^a^	6371 (43.5)	6384 (42.7)	6674 (44.7)	6418 (42.0)	7274 (50.0)	33,122 (44.6)
	Mild	3540 (24.2)	3569 (23.9)	3478 (23.3)	3500 (22.9)	2864 (19.7)	16,951 (22.8)
	Moderate to severe	4616 (31.5)	4905 (32.8)	4661 (31.2)	5233 (34.3)	4309 (29.6)	23,723 (31.9)
	Missing	109 (0.7)	78 (0.5)	133 (0.9)	114 (0.7)	110 (0.8)	544 (0.7)
**Depression (past 2 weeks), n (%)**							
	No depression^a^	7751 (53.0)	7712 (51.6)	7688 (51.4)	7702 (50.5)	7560 (51.9)	38,413 (51.7)
	Mild	3493 (23.9)	3578 (24.0)	3301 (22.1)	3372 (22.1)	3044 (20.9)	16,789 (22.6)
	Moderate to severe	3186 (21.8)	3407 (22.8)	3705 (24.8)	3936 (25.8)	3684 (25.3)	17,918 (24.1)
	Missing	205 (1.4)	239 (1.6)	253 (1.7)	255 (1.7)	269 (1.8)	1221 (1.6)

^a^Referent category for regressions presented in Table 2.

^b^Income was included in the regression in terciles, with the first tercile as the reference group.

^c^Cases with missing responses were excluded from the regression models in Table 2.

^d^Current employment status was not assessed at waves 4 and 5.

^e^N/A: not applicable.

^f^COVID-19 deaths by respondent county were included in the regressions in terciles, with the first tercile as the reference group.

^g^Known COVID-19 deaths and vaccination status were assessed at wave 5 only.

Variables specific to COVID-19 were also collected. Respondents were asked to indicate their level of exposure to COVID-19 in the past 2 months (“tested positive for COVID-19,” “believes had COVID-19 but did not test positive,” “believes someone in their household had COVID-19,” or “does not believe had COVID-19”), stress related to COVID-19 “shelter-in-place” orders, worry about contracting COVID-19, perceived risk of contracting COVID-19 in the next 30 days, and COVID-19-specific coping behaviors (eg, approach coping [broken into low, medium, and high terciles of approach behaviors] and avoidance coping [broken into “any avoidance behaviors” and “no avoidance behaviors]). COVID-19-related coping was assessed using yes/no items based on commonly used measures of coping [7,8]. Exposure to COVID-19-related deaths was calculated using the respondents’ zip code in combination with data from the *New York Times* reporting deaths per 1000 residents to determine low, medium, and high death rates by tercile at each wave. Thus, the level of exposure to COVID-19 deaths was relative to a nationally representative US sample by wave. At wave 5 only, “known COVID-19 deaths” was assessed by asking respondents to indicate how many individuals they personally knew who had died from COVID-19. Levels of categorical COVID-19-related variables and referent categories are displayed in Table 1.

General psychological variables were collected at each wave. Loneliness was assessed with a 3-item scale adapted from the UCLA Loneliness Scale Revised [9], which asked how often respondents feel “lack of companionship,” “left out,” and “isolated from others.” Response options included “hardly ever,” “some of the time,” and “often.” Raw scores ranged from 3 to 9, with scores of 3-5 categorized as “not lonely” and scores of 6-9 categorized as “lonely.” Uncertainty tolerance was assessed with 3 items from the Intolerance of Uncertainty Scale [10], summed and categorized by tercile (low, medium, and high tolerance of uncertainty).

### Analysis

The data included 14,636 interviews conducted on May 11-24, 2020; 14,936 on July 9-22, 2020; 14,946 on October 1-17, 2020; 15,265 on December 4-16, 2020; and 14,557 on March 25-April 13, 2021. Missingness varied by wave in the logistic regressions. Weighted proportions (Table 1) were calculated using R statistical software version 3.6.1. Weighted ordinal logistic regression in SPSS version 27.0 was used to calculate the odds ratios (ORs) for anxiety and depression independently at each wave. Separate wave-by-wave regressions were conducted to test differences in independent variable associations with outcomes across approximately 1 year of the COVID-19 pandemic.

## Results

### Descriptive Statistics

On average, from May 2020 to April 2021, 17,918 of 73,120 (24.1%; n=1221 [1.6%] missing) adults reported moderate-to-severe depression, which increased from waves 1 and 2 (3186-3407 [21.8%-22.8%]) to waves 3 to 5 (3705-3684 [24.8%-25.3%]). On average, 23,723 of 73,796 (31.9%; n=544 [0.7%] missing) reported moderate-to-severe anxiety, with some evidence of decline at wave 5 (waves 1-4=4616-5233 [31.5%-34.3%] vs wave 5=4309 [25.3%]). Descriptive statistics are displayed in Table 1.

### Logistic Regressions

Table 2 displays ORs and 95% CIs from logistic regressions on depression and anxiety at each wave.

**Table 2:** Depression and anxiety full regression models^a^ by wave.

Variable	Depression^b^						Anxiety^b^				
	Wave 1 OR^c^ (95% CI)	Wave 2 OR (95% CI)	Wave 3 OR (95% CI)	Wave 4 OR (95% CI)	Wave 5 OR (95% CI)	Wave 1 OR (95% CI)		Wave 2 OR (95% CI)	Wave 3 OR (95% CI)	Wave 4 OR (95% CI)	Wave 5 OR (95% CI)
Age 18-29 years	*3.57 (3.06-4.18)*	*3.62 (3.10-4.23)*	*4.36 (3.70-5.15)*	*4.39 (3.76-5.11)*	*4.78 (4.01-5.69)*	*2.12 (1.83-2.46)*		*1.72 (1.49-1.99)*	*2.06 (1.76-2.40)*	*2.66 (2.30-3.06)*	*2.78 (2.35-3.28)*
Age 30-44 years	*2.13 (1.82-2.48)*	*2.57 (2.21-3.0)*	*2.63 (2.24-3.10)*	*3.01 (2.59-3.50)*	*2.55 (2.14-3.03)*	*1.98 (1.71-2.29)*		*1.45 (1.26-1.67)*	*2.10 (1.81-2.43)*	*2.13 (1.85-2.45)*	*2.05 (1.74-2.41)*
Age 45-64 years	*1.49 (1.30-1.70)*	*1.64 (1.44-1.88)*	*1.80 (1.55-2.08)*	*1.78 (1.56-2.02)*	*2.00 (1.71-2.33)*	*1.42 (1.25-1.61)*		*1.36 (1.20-1.53)*	*1.61 (1.41-1.83)*	*1.64 (1.46-1.84)*	*1.56 (1.35-1.80)*
Male	1.03 (0.94-1.12)	0.91 (0.84-.99)	0.90 (0.82-.98)	1.0 (0.92-1.09)	0.87 (0.79-.95)	*0.85 (0.78-.92)*		*0.75 (0.70-.82)*	*0.79 (0.72-.85)*	*0.86 (0.79-.93)*	*0.78 (0.71-.85)*
Black	0.81 (0.70-0.93)	0.89 (0.77-1.02)	*0.70 (0.60-0.81)*	1.09 (0.95-1.25)	0.87 (0.75-1.01)	0.88 (0.78-1.01)		0.79 (0.69-0.91)	*0.72 (0.62-0.82)*	0.89 (0.78-1.02)	*0.72 (0.62-0.84)*
Asian/Pacific Islander	*0.59 (0.50-0.70)*	0.79 (0.67-0.94)	0.97 (0.83-1.15)	0.86 (0.73-1.02)	0.84 (0.71-1.0)	0.95 (0.82-1.11)		*1.33 (1.14-1.56)*	*1.40 (1.20-1.63)*	0.91 (0.78-1.06)	1.18 (1.0-1.39)
Hispanic	0.97 (0.86-1.10)	0.82 (0.72-0.93)	0.97 (0.86-1.10)	1.07 (0.95-1.21)	*1.34 (1.18-1.52)*	1.01 (0.90-1.14)		0.96 (0.85-1.09)	0.95 (0.84-1.07)	0.96 (0.85-1.08)	0.90 (0.80-1.02)
Other	1.44 (1.13-1.84)	1.25 (0.99-1.58)	1.25 (0.98-1.59)	*1.56 (1.24-1.97)*	1.31 (1.02-1.68)	1.07 (0.84-1.37)		0.96 (0.77-1.21)	1.08 (0.86-1.37)	1.25 (0.99-1.57)	1.14 (0.89-1.46)
Some college	0.98 (0.88-1.08)	*0.80 (0.72-0.89)*	1.09 (0.99-1.21)	0.85 (0.77-0.94)	*0.82 (0.74-0.91)*	0.93 (0.84-1.03)		1.00 (0.91-1.11)	1.01 (0.92-1.12)	0.95 (0.86-1.04)	*0.81 (0.73-0.90)*
College and above	0.84 (0.75-0.95)	*0.80 (0.71-0.91)*	0.89 (0.78-1.01)	*0.77 (0.69-0.87)*	*0.71 (0.63-0.80)*	0.95 (0.84-1.07)		1.06 (0.95-1.19)	0.90 (0.79-1.01)	0.86 (0.77-0.96)	0.88 (0.78-0.99)
Significant diagnosis	*1.53 (1.40-1.68)*	*1.38 (1.26-1.51)*	*1.57 (1.43-1.72)*	*1.49 (1.36-1.63)*	*1.65 (1.50-1.81)*	*1.33 (1.21-1.45)*		*1.21 (1.11-1.31)*	*1.19 (1.09-1.30)*	1.08 (0.99-1.17)	*1.26 (1.15-1.38)*
2^nd^ tercile income	0.84 (0.76-0.94)	0.97 (0.87-1.08)	*0.77 (0.69-0.86)*	0.95 (0.86-1.06)	0.89 (0.80-0.99)	*0.82 (0.73-0.91)*		0.89 (0.80-0.99)	0.91 (0.82-1.01)	0.97 (0.88-1.08)	*0.82 (0.74-0.91)*
3^rd^ tercile income	*0.78 (0.69-0.88)*	0.88 (0.78-1.00)	*0.70 (0.62-0.80)*	*0.80 (0.71-0.90)*	*0.74 (0.65-0.84)*	*0.72 (0.64-0.81)*		*0.74 (0.66-0.83)*	*0.74 (0.65-0.83)*	*0.81 (0.73-0.91)*	*0.74 (0.65-0.84)*
Going into the workplace	1.10 (0.98-1.23)	0.89 (0.79-0.99)	*0.70 (0.62-0.78)*	N/A^d^	N/A	0.86 (0.76-0.96)		1.12 (1.01-1.24)	0.92 (0.83-1.03)	N/A	N/A
Remote work	1.15 (1.02-1.30)	0.87 (0.77-0.99)	0.90 (0.80-1.03)	N/A	N/A	1.04 (0.93-1.17)		*1.26 (1.12-1.41)*	1.09 (0.96-1.23)	N/A	N/A
Not working (COVID-19)	1.17 (1.03-1.34)	1.26 (1.10-1.46)	1.17 (1.01-1.37)	N/A	N/A	1.16 (1.02-1.32)		*1.28 (1.11-1.46)*	1.03 (0.88-1.19)	N/A	N/A
Not working (other reason)	1.16 (,85-1.60)	1.36 (1.07-1.72)	1.24 (0.98-1.58)	N/A	N/A	1.13 (0.83-1.54)		0.96 (0.76-1.21)	0.97 (0.77-1.22)	N/A	N/A
Lives alone	1.09 (0.96-1.23)	0.99 (0.87-1.12)	0.99 (0.88-1.12)	1.08 (0.96-1.22)	1.23 (1.10-1.39)	0.82 (0.72-0.92)		*0.78 (0.70-0.88)*	1.06 (0.94-1.19)	0.86 (0.77-0.97)	0.85 (0.76-0.96)
Living with children	1.05 (0.95-1.15)	1.04 (0.95-1.14)	*1.28 (1.16-1.41)*	0.94 (0.86-1.03)	1.14 (1.03-1.27)	1.06 (0.97-1.16)		*1.23 (1.12-1.34)*	1.16 (1.06-1.27)	0.91 (0.83-0.99)	0.94 (0.85-1.04)
Democrat	1.10 (1.0-1.21)	0.93 (0.84-1.02)	1.04 (0.94-1.15)	1.16 (1.05-1.27)	1.14 (1.03-1.27)	*1.31 (1.19-1.43)*		1.17 (1.07-1.28)	*1.25 (1.14-1.38)*	*1.21 (1.11-1.33)*	1.07 (0.97-1.18)
Independent	1.16 (1.03-1.31)	1.08 (0.96-1.22)	1.06 (0.93-1.20)	1.01 (0.90-1.15)	1.21 (1.07-1.37)	1.08 (0.96-1.22)		1.17 (1.04-1.31)	1.11 (0.99-1.25)	*1.24 (1.10-1.39)*	1.05 (0.93-1.18)
Rural	1.17 (0.99-1.39)	0.81 (0.68-0.96)	*1.38 (1.16-1.64)*	1.28 (1.08-1.50)	0.95 (0.79-1.14)	0.99 (0.83-1.17)		0.83 (0.71-0.98)	1.20 (1.02-1.42)	1.0 (0.85-1.17)	0.88 (0.74-1.04)
Suburban	1.18 (1.0-1.38)	0.82 (0.69-0.96)	1.28 (1.09-1.51)	1.27 (1.08-1.50)	0.92 (0.77-1.09)	1.01 (0.87-1.19)		1.07 (0.92-1.25)	1.22 (1.04-1.42)	1.21 (1.21-1.03)	0.94 (0.80-1.11)
Urban-suburban	1.23 (1.05-1.43)	0.85 (0.73-0.99)	1.17 (1.0-1.37)	1.13 (0.97-1.32)	1.02 (0.87-1.20)	1.01 (0.87-1.17)		0.95 (0.82-1.09)	1.19 (1.03-1.38)	1.14 (0.98-1.32)	1.06 (0.90-1.23)
Northeast	0.93 (0.80-1.08)	0.93 (0.81-1.06)	0.97 (0.84-1.11)	0.91 (0.80-1.03)	0.83 (0.73-0.96)	1.04 (0.90-1.20)		1.09 (0.97-1.24)	1.23 (1.08-1.40)	0.91 (0.81-1.03)	*0.78 (0.68-0.89)*
Midwest	0.86 (0.75-0.99)	0.93 (0.82-1.06)	1.11 (0.97-1.26)	1.00 (0.88-1.15)	1.08 (0.95-1.24)	0.82 (0.72-0.93)		1.12 (0.99-1.27)	1.04 (0.92-1.18)	0.96 (0.85-1.09)	*0.72 (0.63-0.82)*
South	1.03 (0.92-1.15)	0.88 (0.79-0.98)	0.99 (0.89-1.11)	1.11 (0.99-1.24)	0.96 (0.85-1.08)	0.94 (0.84-1.05)		1.18 (1.06-1.31)	*1.24 (1.11-1.38)*	1.0 (0.90-1.11)	0.95 (0.84-1.06)
Tested positive for COVID-19	*2.50 (1.75-3.58)*	*2.75 (2.13-3.55)*	*3.05 (2.38-3.92)*	*2.59 (2.12-3.17)*	*1.76 (1.47-2.10)*	1.32 (0.92-1.89)		*1.81 (1.40-2.33)*	*1.84 (1.44-2.35)*	*1.54 (1.27-1.88)*	0.96 (0.81-1.14)
Believes had COVID-19	*2.26 (1.89-2.69)*	*1.79 (1.48-2.15)*	*2.38 (1.93-2.93)*	*2.07 (1.75-2.45)*	1.02 (0.83-1.24)	1.08 (0.91-1.30)		0.86 (0.72-1.04)	*2.01 (1.63-2.47)*	*1.57 (1.32-1.86)*	1.12 (0.91-1.37)
Believes household (but not self) had COVID-19	1.08 (0.80-1.46)	1.12 (0.86-1.47)	1.13 (0.85-1.52)	*1.69 (1.34-2.13)*	0.94 (0.76-1.16)	0.65 (0.48-0.88)		0.81 (0.62-1.06)	0.79 (0.59-1.06)	1.28 (1.02-1.62)	0.92 (0.75-1.13)
COVID-19 restriction stress: slight	*1.65 (1.46-1.87)*	*1.41 (1.26-1.58)*	*1.88 (1.68-2.11)*	*1.48 (1.32-1.66)*	*1.60 (1.43-1.79)*	*1.95 (1.75-2.18)*		*1.54 (1.39-1.70)*	*1.61 (1.46-1.78)*	*1.87 (1.69-2.07)*	*1.92 (1.73-2.14)*
COVID-19 restriction stress: moderate	*2.83 (2.48-3.23)*	*2.65 (2.33-3.01)*	*2.84 (2.50-3.24)*	*2.56 (2.26-2.90)*	*2.83 (2.48-3.22)*	*3.77 (3.33-4.28)*		*2.95 (2.62-3.33)*	*3.33 (2.94-3.76)*	*2.78 (2.47-3.13)*	*3.22 (2.83-3.66)*
COVID-19 restriction stress: very	*3.98 (3.36-4.70)*	*3.80 (3.22-4.50)*	*3.74 (3.11-4.50)*	*3.90 (3.31-4.61)*	*3.50 (2.89-4.23)*	*5.63 (4.74-6.69)*		*3.42 (2.88-4.05)*	*3.41 (2.84-4.10)*	*3.44 (2.91-4.06)*	*4.29 (3.53-5.21)*
COVID-19 restriction stress: extreme	*6.24 (4.99-7.80)*	*5.95 (4.74-7.47)*	*6.93 (5.45-8.82)*	*6.75 (5.44-8.39)*	*8.63 (6.68-11.14)*	*6.73 (5.30-8.53)*		*4.33 (3.43-5.48)*	*4.85 (3.84-6.14)*	*4.88 (3.92-6.09)*	*6.62 (5.19-8.45)*
COVID-19 worry: mild	*1.69 (1.50-1.90)*	*1.49 (1.33-1.68)*	*1.61 (1.43-1.81)*	*1.56 (1.39-1.75)*	*1.80 (1.61-2.02)*	*3.37 (3.02-3.76)*		*3.08 (2.76-3.43)*	*2.64 (2.37-2.95)*	*2.98 (2.68-3.33)*	*2.27 (2.03-2.52)*
COVID-19 worry: moderate to severe	*2.48 (2.18-2.82)*	*2.16 (1.89-2.47)*	*2.96 (2.60-3.39)*	*2.38 (2.09-2.70)*	*3.52 (3.09-4.01)*	*6.23 (5.50-7.05)*		*6.39 (5.59-7.19)*	*5.26 (4.63-5.96)*	*5.11 (4.52-5.77)*	*3.40 (2.99-3.86)*
COVID-19 risk: moderately low	1.0 (0.89-1.12)	0.98 (0.87-1.10)	*1.24 (1.10-1.39)*	*1.40 (1.25-1.57)*	0.97 (0.87-1.09)	1.09 (0.98-1.22)		1.05 (0.94-1.18)	1.19 (1.07-1.34)	*1.27 (1.14-1.42)*	1.18 (1.06-1.32)
COVID-19 risk: neither high nor low	1.02 (0.91-1.14)	1.0 (0.89-1.13)	1.09 (0.97-1.21)	1.19 (1.07-1.34)	0.83 (0.73-0.93)	*1.54 (1.38-1.72)*		*1.37 (1.23-1.53)*	*1.38 (1.23-1.54)*	*1.49 (1.34-1.66)*	*1.33 (1.19-1.49)*
COVID-19 risk: moderate or very high	1.15 (0.99-1.33)	1.21 (1.04-1.40)	1.11 (0.95-1.29)	*1.44 (1.24-1.66)*	*0.74 (0.63-0.88)*	*1.39 (1.20-1.62)*		1.27 (1.10-1.46)	1.28 (1.11-1.48)	*1.50 (1.30-1.73)*	*1.35 (1.15-1.59)*
Medium death density	1.08 (0.97-1.20)	1.06 (0.95-1.17)	1.09 (0.97-1.21)	1.15 (1.04-1.26)	1.07 (0.96-1.19)	1.08 (0.97-1.22)		1.0 (0.94-1.14)	0.98 (0.88-1.08)	1.09 (0.99-1.20)	1.0 (0.90-1.11)
High death density	0.97 (0.86-1.09)	1.0 (0.90-1.11)	0.85 (0.76-0.96)	0.88* (0.79-0.98)	0.86 (0.77-0.96)	1.05 (0.93-1.17)		1.11 (1.0-1.23)	*0.80 (0.72-0.89)*	1.05 (0.95-1.17)	0.88 (0.79-0.98)
1 death	N/A	N/A	N/A	N/A	1.08 (0.95-1.22)	N/A		N/A	N/A	N/A	1.08 (0.96-1.22
≥2 deaths	N/A	N/A	N/A	N/A	*1.27 (1.14-1.42)*	N/A		N/A	N/A	N/A	*1.38 (1.24-1.54)*
Fully vaccinated	N/A	N/A	N/A	N/A	1.17 (1.05-1.30)	N/A		N/A	N/A	N/A	0.84 (0.76-0.93)
Partially vaccinated	N/A	N/A	N/A	N/A	1.08 (0.95-1.23)	N/A		N/A	N/A	N/A	*0.79 (0.70-0.89)*
Any loneliness	*3.35 (3.07-3.66)*	*2.98 (2.73-3.26)*	*3.36 (3.07-3.67)*	*2.80 (2.57-3.06)*	*3.60 (3.28-3.95)*	*2.00 (1.83-2.18)*		*2.26 (2.07-2.47)*	*2.11 (1.93-2.31)*	*2.26 (2.07-2.46)*	*2.82 (2.57-3.09)*
Low tolerance of uncertainty	*7.36 (6.49-8.34)*	*8.51 (7.51-9.64)*	*6.84 (6.02-7.77)*	*7.08 (6.26-7.99)*	*6.09 (5.35-6.93)*	*7.23 (6.39-8.18)*		*7.93 (7.02-8.96)*	*7.94 (7.01-8.99)*	*7.84 (6.94-8.84)*	*7.40 (6.53-8.40)*
Medium tolerance of uncertainty	*3.14 (2.83-3.49)*	*3.52 (3.17-3.91)*	*2.73 (2.45-3.04)*	*2.98 (2.69-3.30)*	*2.93 (2.63-3.28)*	*3.03 (2.76-3.33)*		*3.28 (3.0-3.60)*	*2.85 (2.59-3.14)*	*2.67 (2.44-2.93)*	*3.45 (3.11-3.83)*
Any avoidance	*2.31 (2.12-2.52)*	*2.53 (2.32-2.75)*	*2.47 (2.26-2.69)*	*2.27 (2.09-2.46)*	*2.54 (2.32-2.79)*	*1.43 (1.32-1.55)*		*1.30 (1.19-1.41)*	*1.39 (1.28-1.52)*	*1.46 (1.35-1.59)*	*1.45 (1.33-1.59)*
Low approach coping	*1.67 (1.51-1.85)*	*1.78 (1.61-1.97)*	*1.84 (1.66-2.04)*	*1.58 (1.43-1.75)*	*2.19 (1.96-2.45)*	0.99 (0.90-1.09)		1.13 (1.03-1.24)	0.99 (0.89-1.09)	1.01 (1.35-1.59)	0.92 (0.82-1.02)
Medium approach coping	1.15 (1.04-1.28)	1.19 (1.07-1.32)	*1.24 (1.11-1.39)*	*1.45 (1.31-1.61)*	*1.65 (1.46-1.87)*	0.92 (0.84-1.02)		1.09 (0.99-1.21)	0.94 (0.84-1.04)	1.02 (0.93-1.13)	0.93 (0.83-1.04)

^a^Each column presents ORs (95% CIs) from separate regression models (for referent categories, see Table 1). *N* varied by model.

^b^Italicized OR (95% CI) values signify *P*<.001.

^c^OR: odds ratio.

^d^N/A: not applicable.

#### Sociodemographic Variables

Of the sociodemographic variables, a younger adult age evidenced the strongest associations with depression and anxiety across waves. The effect of a younger age (ie, age 18-29 years) on depression was nearly double that for anxiety (waves 1-5 depression ORs 3.57-4.78, all *P*<.001, vs waves 1-5 anxiety ORs 2.12-2.78, all *P*<.001). Respondents aged 18-29 years and aged 30-44 years evidenced increasing moderate-to-severe depression rates from wave 1 to wave 5 (age 18-29 years=5459-6871 [37.3%-47.2%]; age 30-44 years=4069-4673 [27.8%-32.1%-), while older age groups had stable or declining rates (age 45-64 years=2386-2576 [16.3%-17.7%]; age ≥65 years=1171-815 [8.0%-5.6%]). To explore why younger adults might be more prone to persistent depressive symptoms, post hoc analyses tested interactions of age (continuous) with COVID-19-specific and psychological variables of depression. Tests of interactions did not identify any variable consistently related more strongly to greater depression in younger relative to older adults.

Women reported more anxiety than men (waves 1-5 ORs 1.18-1.28, all *P*<.001). Being in the highest income tercile was associated consistently with lower depression and anxiety (waves 1-5 depression ORs 0.78-0.74, all *P*<.001; waves 1-5 anxiety ORs 0.72-0.74, all *P*<.001). Medical comorbidity was related to depression and anxiety at most waves, although effects were not large (waves 1-5 depression ORs 1.53-1.65, *P*<.001; waves 1-5 anxiety ORs 1.33-1.26, all *P*<.001). Other sociodemographic variables were not associated consistently with outcomes.

#### COVID-19-Specific Variables

Of the COVID-19-specific variables, perceived stress from COVID-19 restrictions evidenced the strongest, graded relationships with depression (waves 1-5 “slightly stressful” to “extremely stressful” ORs from 1.65-1.48 to 6.24-8.63, all *P*<.001) and anxiety (waves 1-5 “slightly stressful” to “extremely stressful” ORs from 1.95-1.92 to 6.73-6.62, all *P*<.001) across waves. COVID-19-related worry also evidenced a strong, graded relationship with anxiety, which diminished at wave 5 (waves 1-5 “mild” to “moderate to severe” ORs from 3.37-2.27 to 6.23-3.40, all *P*<.001); its relationship with depression was somewhat weaker. Testing positive for COVID-19 in the past 2 months (or believing one had COVID-19) was associated consistently with higher depression. Perceived COVID-19 risk was associated with higher anxiety, with small effects (waves 1-5 “moderately to very high” ORs 1.39-1.35, all *P*<.001). Knowing 2 or more people (vs knowing no one) who had died from COVID-19 (measured only at wave 5) was associated with both outcomes, with small effects (wave 5 depression OR=1.27, *P*<.001; wave 5 anxiety OR 1.38, *P*<.001). At wave 5, being partially vaccinated (vs no vaccination) was associated with less anxiety, with small effects (wave 5 OR 0.79, *P*<.001). With regard to COVID-19-related coping, reporting any (vs no) avoidance behaviors was associated consistently with more depression and anxiety, with small-to-moderate effect sizes, which were greater for depression than anxiety (waves 1-5 depression ORs 2.31-2.54, all *P*<.001; waves 1-5 anxiety ORs 1.43-1.45, all *P*<.001). Lower approach-oriented coping was associated consistently with greater depression (but not anxiety) across waves, with small effect sizes (waves 1-5 “low approach” depression ORs 1.67-2.19, all *P*<.001).

#### Psychological Variables

Of the general psychological variables, lower tolerance of uncertainty evidenced the strongest, graded relationships with depression (waves 1-5 “low” to “medium” ORs from 7.36-6.09 to 3.14-2.93, all *P*<.001) and anxiety (waves 1-5 “low” to “medium” ORs from 7.23-7.40 to 3.03-3.45, all *P*<.001). Respondents reporting any (vs no) loneliness also reported more depression (waves 1-5 ORs 3.35-3.60, all *P*<.001) and anxiety (waves 1-5 ORs 2.00-2.82, all *P*<.001) across waves, with moderate-to-large effect sizes, which were slightly larger for depression than anxiety.

## Discussion

### Principal Findings

Findings from 5 waves of large, nationally representative samples provided substantial evidence that the US population has experienced increased rates of clinically relevant depression and anxiety in response to the onset of the COVID-19 pandemic, which have been sustained across the majority of the first year of the pandemic. Rates of moderate-to-severe depression (n=17,918, 24.1%) and anxiety (n=23,723, 31.9%) were much higher than documented prepandemic levels of depression (n=7%) [11] and anxiety (6.1%) [12]. Logistic regression analyses revealed that in general, the magnitude of associations of sociodemographic and other variables with mental health outcomes did not evidence a consistent pattern of change over the year. Of the sociodemographic variables, age was most robustly and reliably associated with the outcomes. Consistent with other research, younger adults (age<44 years) demonstrated a substantially higher likelihood of reporting moderate-to-severe depression compared to adults ≥65 years old [13-15]. These findings contribute to the literature by demonstrating that the risk for depression to younger adults persisted late into the pandemic, whereas older adults began to decline in depression and anxiety. These findings have important implications for mental health both now and in the future. MDD is episodic in nature, and a documented risk factor for recurrent episodes is the frequency and duration of prior episodes [16]. Promoting mental health awareness and psychoeducation will be crucial to reaching young adults, as will making mental health care easily accessible through integration with primary care and leveraging technology to deliver remote care. Research is needed to identify novel methods to reach younger adults to assess for mental well-being as well as deliver mental health care that is sensitive to and able to address specific generational differences in the experience of the pandemic that may contribute to worse mental health outcomes [17].

Over and above sociodemographic factors, the strongest and most persistent COVID-19-related factors related to the outcomes were testing positive for COVID-19 (for depression), perceiving stress from pandemic-related restrictions, worry, and coping behaviors related to the pandemic. Presumably, the COVID-specific factors contributing to mental health outcomes will become less relevant as the pandemic wanes, with the exception of potentially chronic effects of having the disease. However, even in the light of efforts to manage the pandemic through vaccination and ongoing implementation of mask recommendations/mandates, COVID-19 continues to be diagnosed in the vaccinated and especially the unvaccinated, and research has emerged related to effects of long COVID-19 [18]. Thus, COVID-19-related stress, worry, and coping behaviors continue to be significant factors in COVID-19-related depression and anxiety that warrant long-term monitoring. Specifically, individuals who have been diagnosed with COVID-19 warrant long-term monitoring for symptoms of depression.

Among general psychological factors, a lower tolerance of uncertainty was the most potently and consistently associated with outcomes. Loneliness was also associated with a greater likelihood of moderate-to-severe depression and anxiety. Loneliness has been identified by numerous studies as an increasing contributor to mental health outcomes, such as depression and anxiety, as well as an indicator of diminished quality of life per se, especially following the onset of the COVID-19 pandemic. Evidence also suggests that interventions designed to improve social connection behaviorally and challenge patterns of thinking that contribute to loneliness (ie, cognitive behavioral therapy [CBT]) are effective at reducing perceived loneliness and associated depressive symptoms [19,20]. Recent research has explored digital applications of these CBT principles, a delivery method that is recognized as critical to intervention dissemination, particularly with the necessity of remote delivery of services in the context of the COVID-19 pandemic [21]. Similarly, CBT-based interventions for intolerance of uncertainty should also be emphasized and disseminated, given present findings. For example, mindfulness-based interventions that promote tolerance of psychological experiences [22] and CBT-based interventions that promote adaptive coping and disconfirmation of feared outcomes may be beneficial for individuals with chronic intolerance of uncertainty [23,24].

### Conclusion

In summary, data from large, nationally representative samples of adults collected at 5 waves over a year’s period reveal that symptoms of depression and anxiety are markedly elevated from shortly after COVID-19 was first diagnosed in the United States through more than 1 year later. Health care professionals should monitor at-risk groups, particularly younger adults, adults who evidence intolerance of uncertainty or loneliness, and those who have had the disease. This study identified both vulnerable groups and psychological processes that can be targeted to promote the psychological health of the population as the nation continues to move through profoundly challenging times.

## Abbreviations

CBT: cognitive behavioral therapy
MDD: major depressive disorder
OR: odds ratio
PHQ-8: Patient Health Questionnaire-8
PROMIS: Patient-Reported Outcome Measurement Information System
UCLA: University of California, Los Angeles
